# Supplementary figures and images for: CryoEM structure of the Nipah virus nucleocapsid assembly
Source: PLoS Pathog. 2021 Jul 16;17(7):e1009740. doi: 10.1371/journal.ppat.1009740 (PMC8318291; doi:10.1371/journal.ppat.1009740)

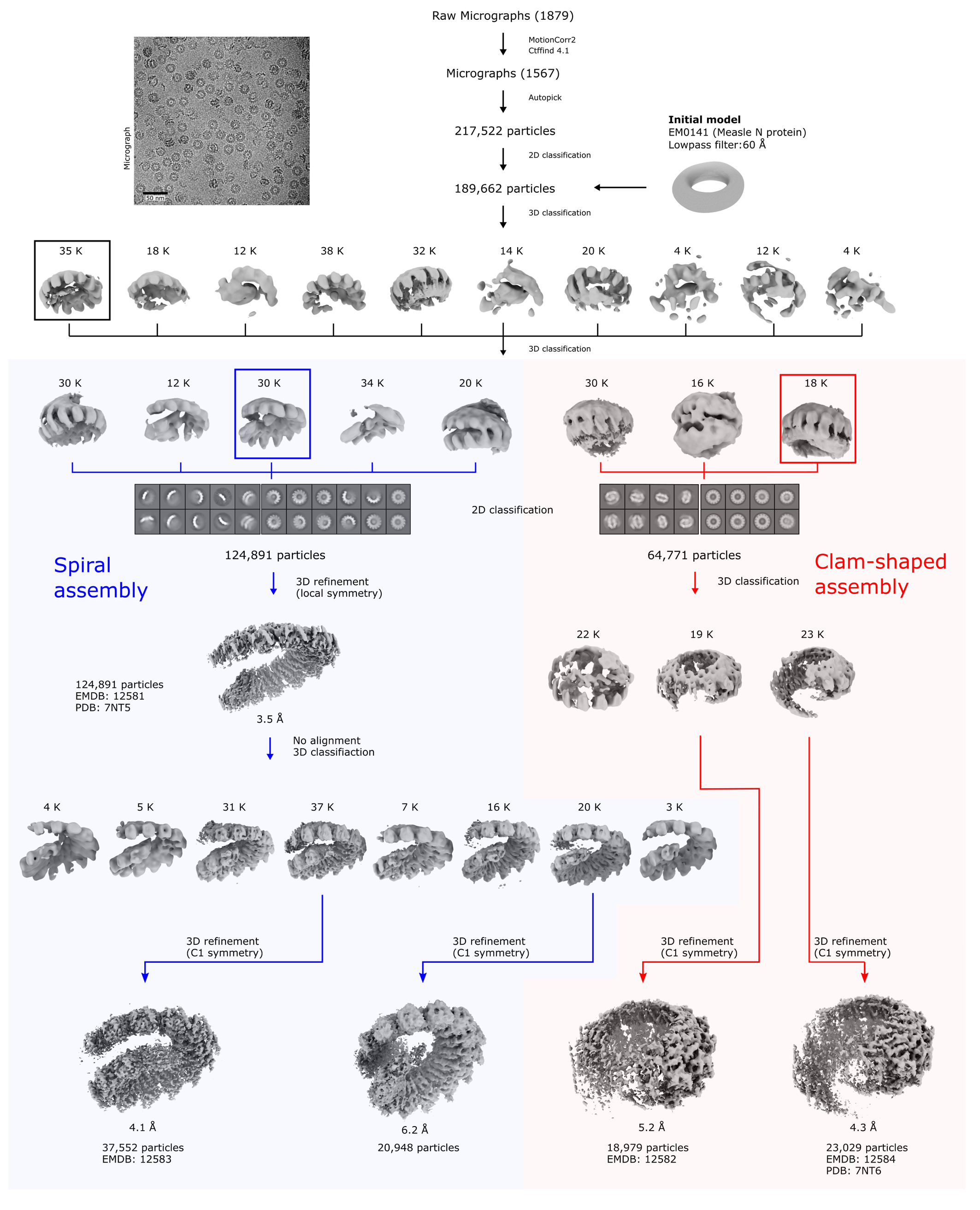

Supplement: S1 Fig — After the 2D classification, 189,662 particles were initially sorted by 3D classification using an initial model generated from EMDB-0141 low-pass filtered to 60 Å[22]. The best 3D class (squared on the figure) was used as a reference for a new round of 3D classification, to sort the 189,662 particles into the spiral assembly (65%) and clam-shaped assembly (35%) groups. An additional 2D classification was performed to inspect the selected particles and 2D class averages for each type of assembly. For the spiral assembly, a mask representing a single turn of spiral assembly was applied to the map, leading to 3.5 Å structure. Further 3D classification without alignment resulted in several spiral assembly maps, with different conformations and compositions (S3B Fig). For the clam-shaped assembly, further 3D classification resulted in semi-spiral clam shaped assembly and in a spiral clam shaped assembly. (TIF) [file ppat.1009740.s001.tif]

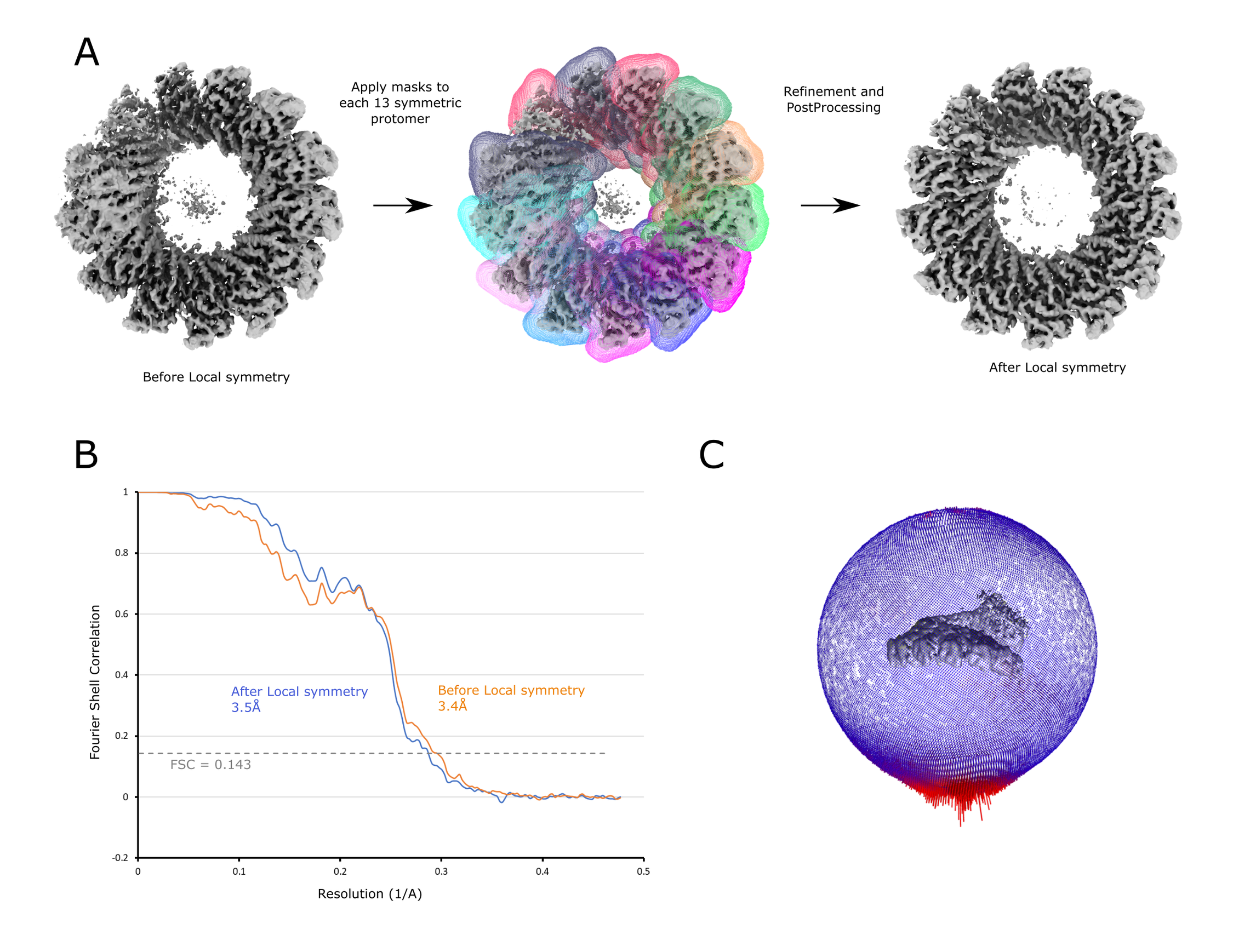

Supplement: S2 Fig — (A) Local symmetry refinement workflow. (B) “Gold-standard” FSC plot before and after local symmetry refinement. (C) Angular distribution plot for the spiral assembly. (TIF) [file ppat.1009740.s002.tif]

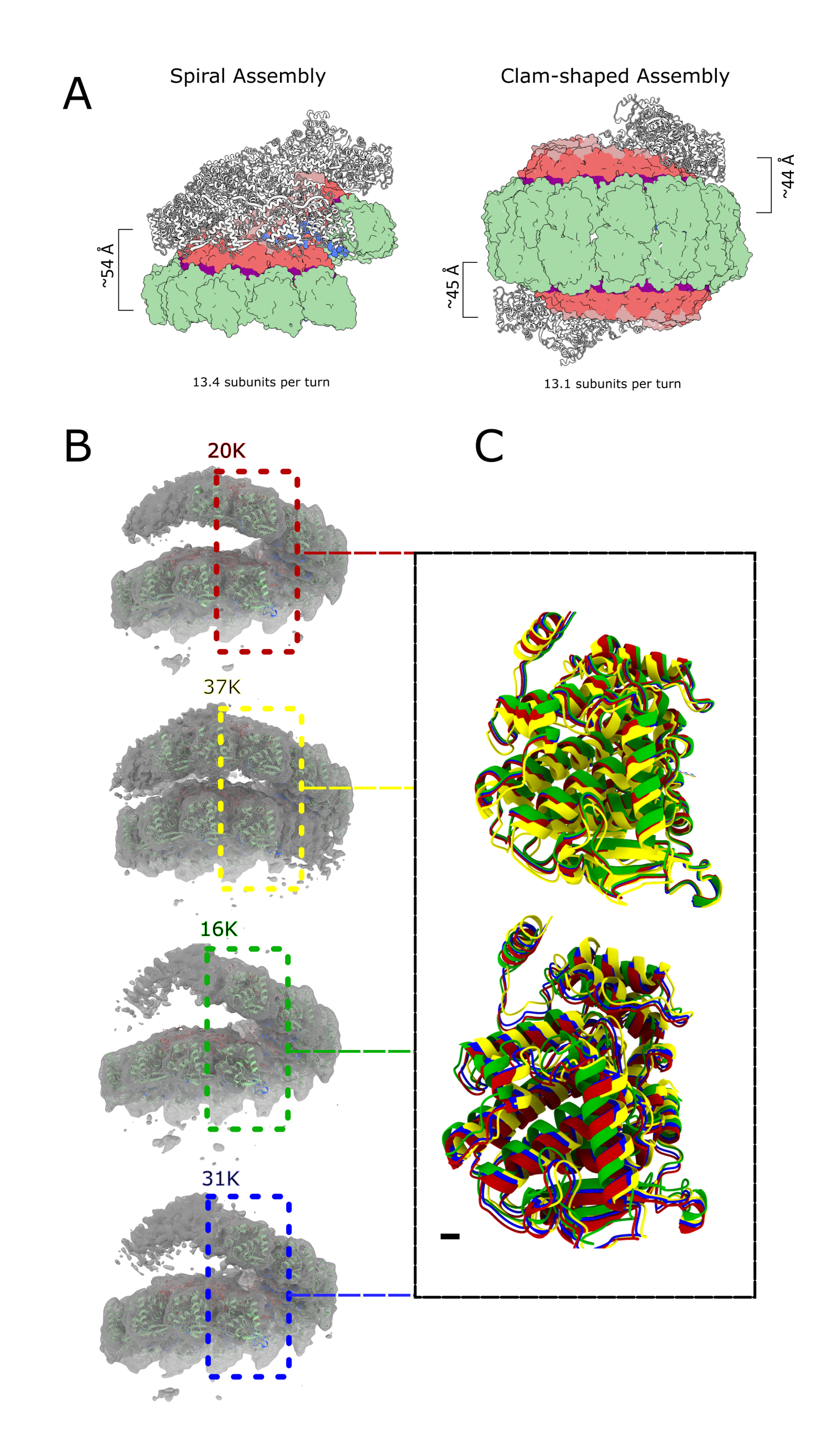

Supplement: S3 Fig — (A) Ribbon model in white is a duplicate of the helical assembly, generated from the original structure, aligned using Chimera MatchMaker feature, so that its first subunit matches the last subunit of the original structure. The resulting spiral turn was used for calculation of the pitch and the number of subunits per turn. (B) CryoEM maps of the top four 3D classes from the classification of NiV N protein helical assembly (S1 Fig), shown along with ribbon diagrams of fitted N protein subunits. Number of particles contributing to each respective class is indicated above each model. Overlay of all the fitted NiV N protein models reveals a subtle variation in the seam region of the helical turn. (C) Overlay of single subunits from the four different assemblies shown in (B), calculated and shown for two single subunits of each subunit, taken from two different positions of the helical assembly. Scale bar, 1 Å. (TIF) [file ppat.1009740.s003.tif]

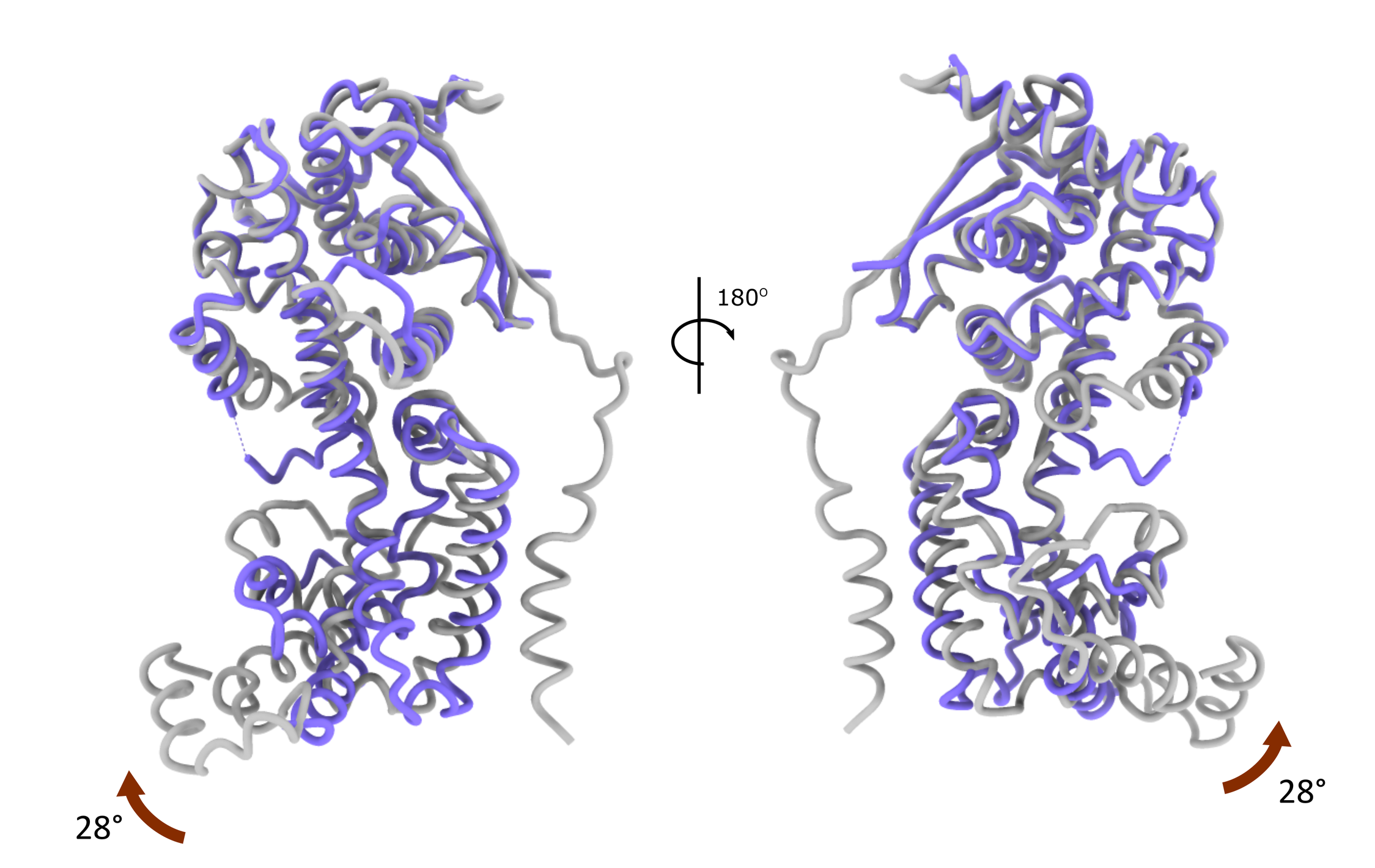

Supplement: S4 Fig — Superimposed models are presented as cartoons. The RNA-free N protein (pdb:4co6)[15] is in light purple; while the RNA-bound protein is in light grey. (TIF) [file ppat.1009740.s004.tif]

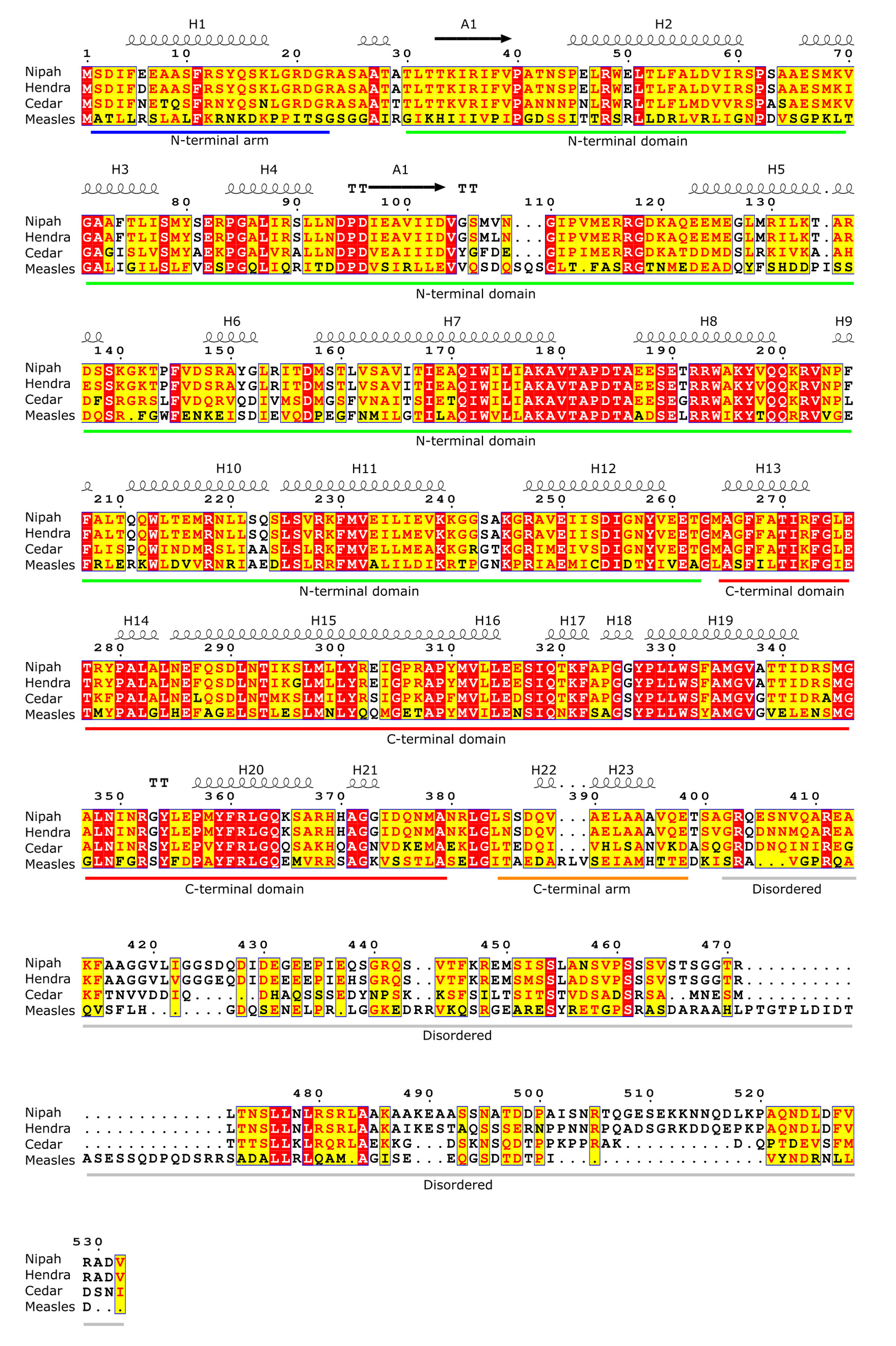

Supplement: S5 Fig — Multiple alignment graphic was prepared using ESPript 3.0 (http://espript.ibcp.fr/ESPript/cgi-bin/ESPript.cgi). (TIF) [file ppat.1009740.s005.tif]

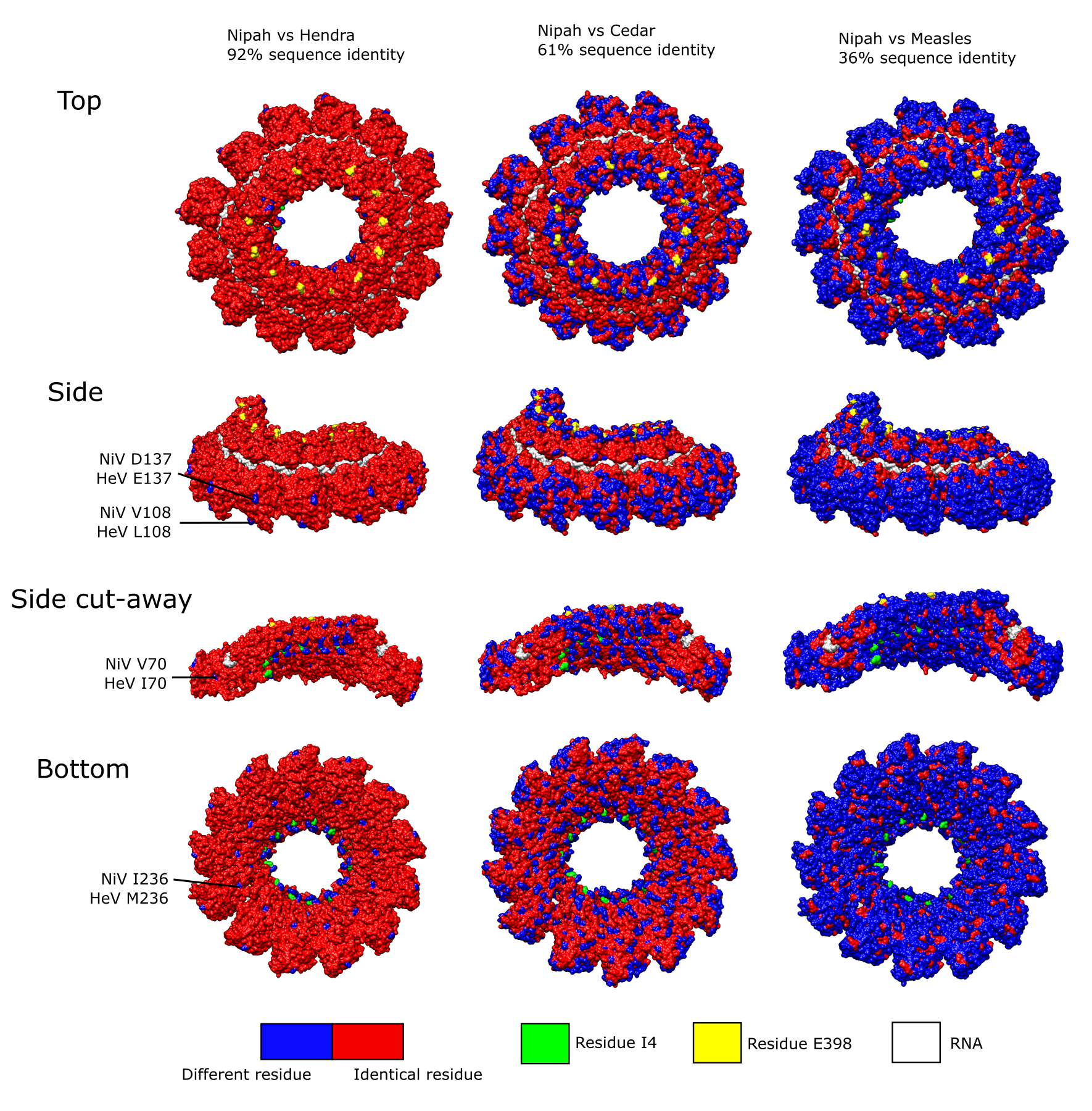

Supplement: S6 Fig — Amino acids differing between the NiV and HeV N proteins, are labelled. (TIF) [file ppat.1009740.s006.tif]

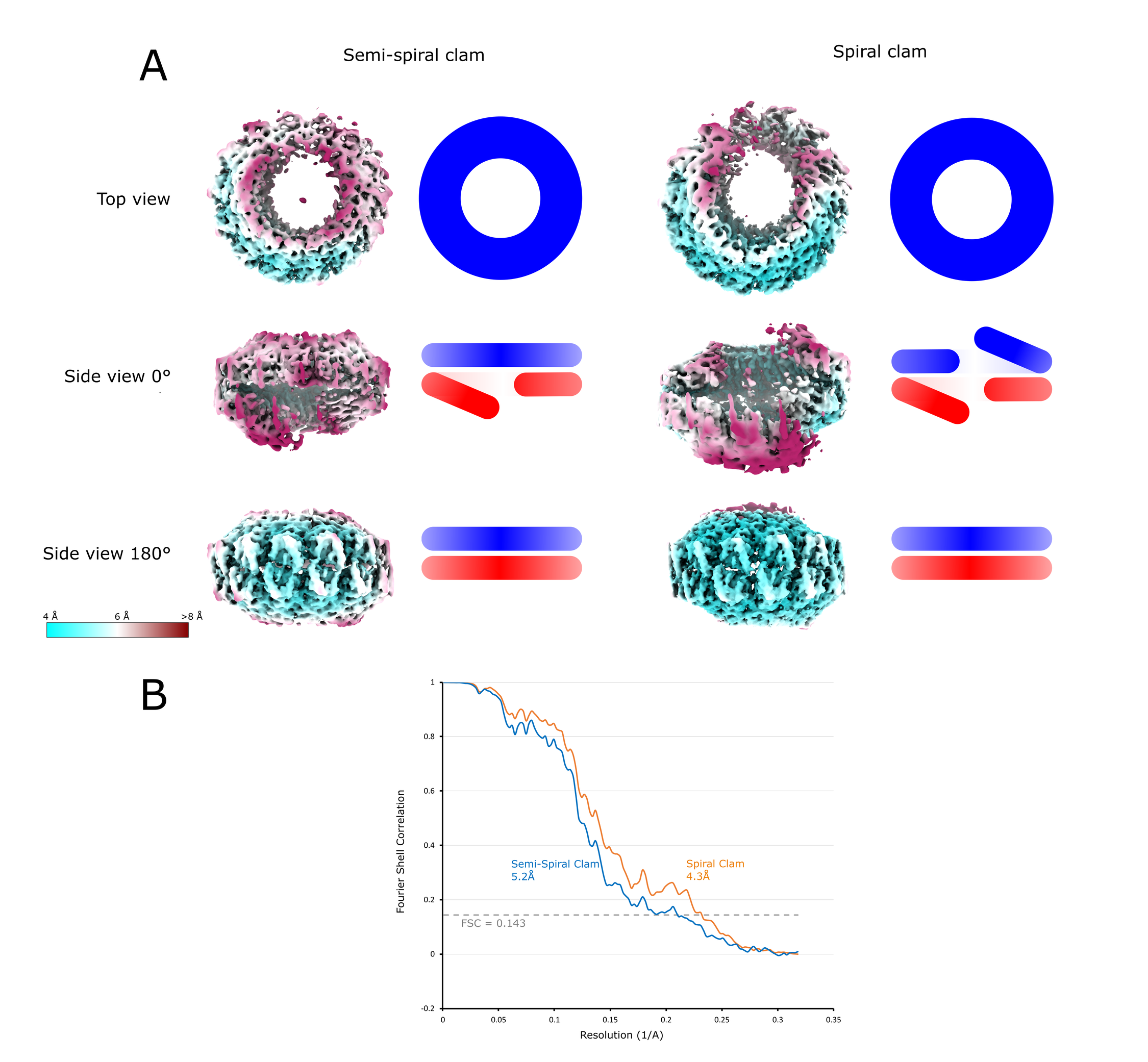

Supplement: S7 Fig — (A) Local resolution of CryoEM maps for each of the two assemblies are shown in three different views. Cartoon illustrations for each view are also presented. (B) “Gold-standard” FSC plots for the semi-spiral clam and spiral clam-shaped assembly. (TIF) [file ppat.1009740.s007.tif]

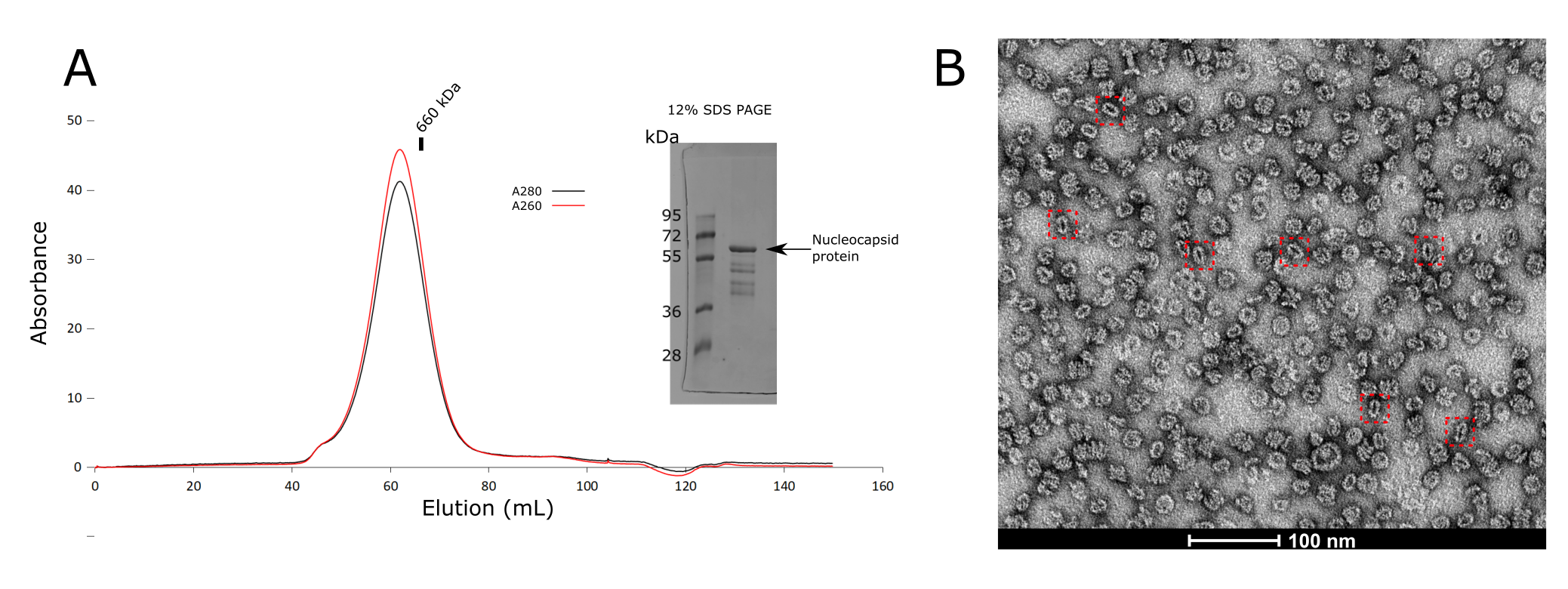

Supplement: S8 Fig — (A) SEC purification and SDS PAGE profile of Nipah N protein. Elution retention volume of thyroglobulin is indicated. (B) Negative stained EM micrograph of the SEC purified N protein from (A). Dashed red boxes represents side-views of the clam-shaped assembly. (TIF) [file ppat.1009740.s008.tif]

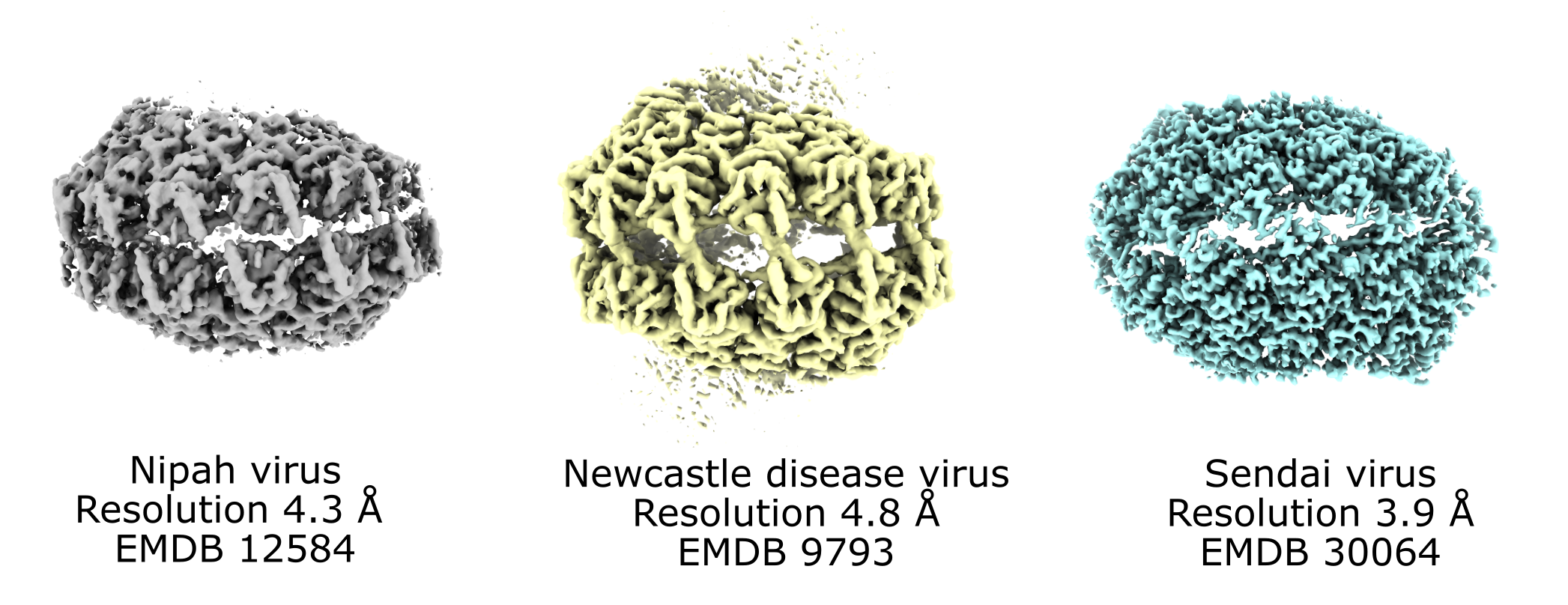

Supplement: S9 Fig — (TIF) [file ppat.1009740.s009.tif]

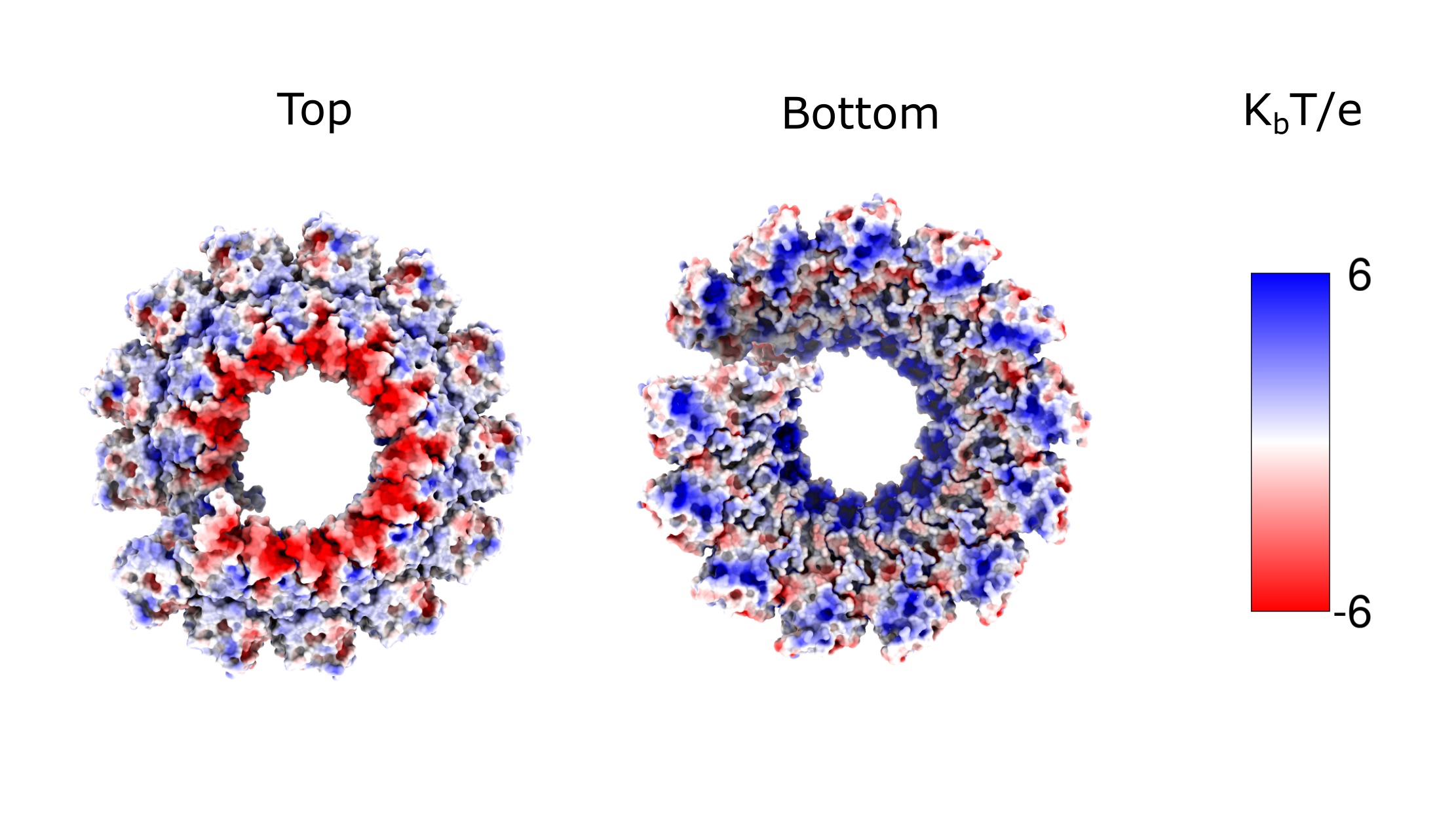

Supplement: S10 Fig — Calculations were performed at pH 7.0 and 150 mM salt concentration. Positive and negative charges are colored in blue and red, respectively. (TIF) [file ppat.1009740.s010.tif]
