# Supplementary material for: CryoEM structure of the Nipah virus nucleocapsid assembly
Source: PLoS Pathog. 2021 Jul 16;17(7):e1009740. doi: 10.1371/journal.ppat.1009740 (PMC8318291; doi:10.1371/journal.ppat.1009740)
Supplement: S1 Table — The buried surface area was calculated for the helical assembly of each virus. (DOCX) [file ppat.1009740.s011.docx]

|  | NiV (This study) | PIV5 (PDB:4xjn) | MeV (PDB:6h5q) |
| --- | --- | --- | --- |
| Interface area (A^2^) | 2998 (100%) | 2918 (100%) | 2931 (100%) |
| Polar interface area (Å^2^) | 2011 (67%) | 1931 (66%) | 1812 (62%) |
| Nonpolar interface area (Å^2^) | 986 (33%) | 987 (34%) | 1119 (38%) |
