# Supplementary material for: CryoEM structure of the Nipah virus nucleocapsid assembly
Source: PLoS Pathog. 2021 Jul 16;17(7):e1009740. doi: 10.1371/journal.ppat.1009740 (PMC8318291; doi:10.1371/journal.ppat.1009740)
Supplement: S2 Table — Rotational and translational values were derived from comparison of the RNA-free (pdb:4co6)[15] and RNA-bound states. All values were estimated as described in Material and Methods. (DOCX) [file ppat.1009740.s012.docx]

| Rotation Angle (°) | 27.9 |
| --- | --- |
| Translation (Å) | 1.8 |
| Closure (%) | 46.8 |
| Hinge region residues | 263-265  303-304  317-321 |
