# Supplementary material for: CryoEM structure of the Nipah virus nucleocapsid assembly
Source: PLoS Pathog. 2021 Jul 16;17(7):e1009740. doi: 10.1371/journal.ppat.1009740 (PMC8318291; doi:10.1371/journal.ppat.1009740)
Supplement: S3 Table — The genus for each virus is indicated in brackets. (DOCX) [file ppat.1009740.s013.docx]

|  | NiV | HeV | CeV | NarV | BeiV | RPV | MV | PIV5 |
| --- | --- | --- | --- | --- | --- | --- | --- | --- |
| NiV (*Henipavirus*) |  |  |  |  |  |  |  |  |
| HeV (*Henipavirus*) | 92.1 |  |  |  |  |  |  |  |
| CeV (*Henipavirus*) | 59.4 | 59.8 |  |  |  |  |  |  |
| NarV (*Narmovirus*) | 37.2 | 37.4 | 34.9 |  |  |  |  |  |
| BeiV (*Jeilongvirus*) | 33.7 | 33.6 | 34.9 | 39.6 |  |  |  |  |
| RPV (*Morbillivirus*) | 33.5 | 33.0 | 32.1 | 37.5 | 35.9 |  |  |  |
| MV (*Morbillivirus*) | 32.1 | 32.5 | 32.8 | 38.7 | 36.3 | 74.5 |  |  |
| PIV5 (Orthorubulavirus) | 27.9 | 27.7 | 28.1 | 28.5 | 26.4 | 27.3 | 26.3 |  |
| NDV (Orthoavulavirus) | 28.8 | 28.7 | 28.3 | 26.6 | 27.2 | 25.2 | 25.5 | 34.6 |
