# Supplementary material for: CryoEM structure of the Nipah virus nucleocapsid assembly
Source: PLoS Pathog. 2021 Jul 16;17(7):e1009740. doi: 10.1371/journal.ppat.1009740 (PMC8318291; doi:10.1371/journal.ppat.1009740)
Supplement: S4 Table — (DOCX) [file ppat.1009740.s014.docx]

|  | Spiral | Spiral Clam |
| --- | --- | --- |
| **Data collection**  Voltage (kv)  Detector  Electron exposure (e-/Å^2^)  Defocus range (µm)  Pixel size (Å) | 300  Gatan K2 Summit  41.2  0.5 to 2.1  1.048 | |
| **Data processing**  Symmetry imposed  Final particle images (no.)  Map resolution (Å)  FSC threshold  Map sharpening B factor (Å^2^) | C1  124,891  3.5  0.143  -69 | C1  23,029  4.3  0.143  -10 |
| **Model composition**  Non-hydrogen atoms  Protein residues  Nucleic acid residues | 40200  5088  78 | 24128  5532  90 |
| **Validation**  RMS Bond lengths (Å)  RMS Bond angles (°)  MolProbity score  Clashscore  Rotamer outliers (%)  Ramachandran Favored (%)  Ramachandran Outliers (%)  RNA average suiteness | 0.013  2.2  1.7  6.53  1.5  96.5  0.18  0.560 | 0.013  1.9  1.1  0.87  0.0  95.6  0.33  0.566 |
