# Supplementary material for: CryoEM structure of the Nipah virus nucleocapsid assembly
Source: PLoS Pathog. 2021 Jul 16;17(7):e1009740. doi: 10.1371/journal.ppat.1009740 (PMC8318291; doi:10.1371/journal.ppat.1009740)
Supplement: S5 Table — (DOCX) [file ppat.1009740.s015.docx]

| Region | RMSD (Å) |
| --- | --- |
| Whole protein (residue 32-369) | 3.3 |
| N-terminal Ncore (residue 32-258) | 1.7 |
| C-terminal Ncore (residue 286-369) | 1.7 |
