# Supplementary material for: CryoEM structure of the Nipah virus nucleocapsid assembly
Source: PLoS Pathog. 2021 Jul 16;17(7):e1009740. doi: 10.1371/journal.ppat.1009740 (PMC8318291; doi:10.1371/journal.ppat.1009740)
Supplement: Attachment — Submitted filename: Nipah_Response_to_reviewers.docx [file ppat.1009740.s016.docx]

Dear Editors,

Following your recent correspondence, we submit a revision of the manuscript by Ker *et al.* " CryoEM structure of the Nipah virus nucleocapsid assembly", Ref: PATHOGENS-D-21-00703.

**Detailed point-by-point response to the reviewers’ comments**

We would like to thank Referees for critically reading the manuscript and for making useful suggestions.

Reviewer #1 (*all comments in italics*):

*I have only some minor comments that may be used by the authors to improve the discussion.
In the discussion, the authors wrote: “Most strikingly, some viruses, like Rabies, a member of the Rhabdoviridae, bind the RNA inside the helicoidal assembly (45) in contrast to the viruses of the Paramyxoviridae (e.g. NiV), Filoviridae(42, 46) and Pneumoviridae(47, 48) families, where the RNA is wrapped around the outside."
First, reference 45 is related to VSV N. The correct reference to rabies virus N is Albertini et al. (Science. 2006 Jul 21;313(5785):360-3.) which has to be quoted too.
However, those two structures of rhabdovirus nucleoproteins are obtained from crystal rings. In the virions, in the RNP assembly, the proteins are tilted (compared to their organization in the rings) and thus the RNA is not really positioned inside the helicoidal assembly. See Ge et al. (Science. 2010 Feb 5;327(5966):689-93) for VSV and Riedel et al. (Sci Rep. 2019 Jul 3;9(1):9639) for rabies virus.
Interestingly, reminiscent of the organization of clam-like assemblies of NIpah N, in the asymmetric unit or rabies virus nucleoprotein crystal structure (PDB code: 2GTT), there are two rings, made of 11 nucleoproteins, that are associated head to head through contacts involving their N-terminal lobes.*

We thank the Referee for bringing up this important point. We have added the correct references and rephrased the statement to better reflect the fact that the bound RNA in members of the Rhabdoviridae is not entirely positioned inside the nucleocapsid assembly.

The new statement reads as follows:

“Interestingly, crystal structures of circular assemblies of the rabies virus and vesicular stomatitis virus (46, 47), members of *Rhabdoviridae* family, indicated that RNA-binding groove faces towards the inside of the central tunnel (13). However, in the helical assembly in virions (48, 49) the individual N proteins are tilted, compared to their organization in the rings and the RNA is not positioned inside the helicoidal assembly, although it does bind closer to the central axis than in the viruses of the *Paramyxoviridae* (e.g. NiV), *Filoviridae*(43, 50) and *Pneumoviridae*(51, 52) families, where the RNA-binding grove is located closer to the outer edge of the helical assembly.”

*Second, when comparing rhabdovirus and paramyxovirus, it might be pertinent to discuss the N0P structures. For paramyxovirus, as mentioned by the authors, the P-protein segment is bound in a groove which is occupied by the N-terminal helix of one adjacent subunit and a C-terminal region of another adjacent subunit. However, for rhabdoviruses, the N-terminal part of P occupies the RNA binding site (Leyrat et al. PLoS Pathog. 2011 Sep;7(9):e1002248.). Therefore, the way the N is kept soluble and RNA-free by P is slightly different for paramyxoviruses and rhabdoviruses.*

This is again very useful suggestion. We have added a sentence in the discussion to reflect this point:

“It is interesting to note, that compared to *Paramyxoviridae* and *Pneumoviridae*, *Rhabdoviridae* also adopt a different strategy to inhibit non-specific RNA binding of its N protein, by inserting the P protein directly into the RNA binding groove (15, 53, 54). “

Reviewer #2:

*1) While describing the symmetry of the nucleocapsid helices, the authors call it helicoid, helicoidal or helical. I would suggest to stick to the conventional term, helical, throughout the manuscript.*

As suggested, we have now used the term “helical” throughout the whole manuscript.

*2) In the second paragraph of the introduction, N is introduced as nucleocapsid (N) protein. It should be nucleoprotein.*

We have corrected this.

*3) I would suggest to change the word order in the following sentence (page 2) to make it clearer: ‘The only available structural information for the NiV nucleocapsid is for a truncated single subunit lacking N-terminal (residues 1-31) and C-terminal (residues 384-532) regions, which was determined in complex with a 50 amino acid peptide of the P protein(15).’ => ‘The only structural information for the NiV nucleocapsid is available for a truncated single subunit lacking N-terminal (residues 1-31) and C-terminal (residues 384-532) regions, which was determined in complex with a 50 amino acid peptide of the P protein(15).’*

We have changed the word order as suggested.

*4) Would it be possible to change the color of the C-terminal arm so that the reader can more easily see it in the figures? In the present version, the colors or the C-terminal domain of the Ncore and of the C-terminal arm are not easy to distinguish, and in particular in the Figure 1 one would like to be able to clearly see the position of the C-terminal arm that, in the nucleocapsid helix, should be situated between two successive helical turns.*

As suggested, we have changed the colour (to light grey) to improve clarity .

*5) The current discussion about the possible relevance of the clam-shaped assembly is very interesting. In addition, a very recent paper (Zhang et al., Communications Bioloby 2021) describes the structure of the double-headed nucleocapsids from Sendai virus and presents a map of the clam-shaped structure which is better resolved than the one of the Nipah virus. It seems important to compare the two structures (the map is deposited in the EMDB) and to enrich the discussion by including the new information offered in the Sendai virus work.*

As suggested, we have cited the latest work from Zhang et al. (Communication Biology, 2021) in the discussion and compared the cryoEM maps of clam-shaped assemblies (Figure S9).

*6) Please check the legend of the Figure S3A and state what exactly is on the left and on the right.*

We have added labels to indicate what is on the left and on the right.

In addition to changes requested by Referees, we have carefully read the manuscript and made several further amendments to improve clarity. We have also indicated final, improved, statistics for the refined model of the Cryo-EM reconstruction (following deposition and validation).
